# In Vitro Fertilization in Kazakh Whiteheaded Cattle: A Comparative Study

**DOI:** 10.3390/life13081632

**Published:** 2023-07-27

**Authors:** Bolat Seisenov, Dulat Duimbayev, Nurlybay Kazhgaliyev, Talgat Abdrakhmanov, Alexandra Tegza, Rustem Abeldinov, Nadezhda Burambayeva, Alma Temirzhanova, Ivan Tegza, Zhomart Kemeshev, Assylbek Zhanabayev, Nurbolat Akhmetbekov, Marat Aisin, Kuandyk Zhugunissov, Arman Issimov

**Affiliations:** 1 Assyl Tulik, Republican Center for Livestock Breeding, Astana 010000, Kazakhstan; 2 Department of Animal Husbandary and Bioresourses, Zhangir Khan West Kazakhstan Agrarian—Technical University, Oral 090000, Kazakhstan; dulat_18@mail.ru; 3 Faculty of Veterinary and Animal Husbandary Technology, Saken Seifullin Kazakh Agrotechnical University, Astana 010000, Kazakhstan; kazhgaliev.n@mail.ru (N.K.); talgat.abd@mail.ru (T.A.); zhomart-naiman@mail.ru (Z.K.); zhanabaev.asylbek@mail.ru (A.Z.); akhmetbec@mail.ru (N.A.); 4 Department of Veterinary Medicine, A. Baitursynov Kostanay Regional University, Kostanay 110000, Kazakhstan; tegza.4@mail.ru (A.T.); tegza4@mail.ru (I.T.); aisin-m65@mail.ru (M.A.); 5 Department of Zootechnology, Genetics and Breeding, Toraighyrov University, Pavlodar 140000, Kazakhstan; abrustem@mail.ru (R.A.); nadezhdaburambaeva@gmail.com (N.B.); alma.temirzhanova.74@mail.ru (A.T.); 6 Research Institute for Biological Safety Problems, Gvardeiskiy 080409, Kazakhstan; 7 Department of Biology, K. Zhubanov Aktobe Regional University, Aktobe 030000, Kazakhstan

**Keywords:** IVF, embryo, Kazakh Whiteheaded cattle

## Abstract

In vitro fertilization (IVF) technologies have great potential in the preservation of endangered species. In the current study, an IVF experiment was carried out to evaluate whether reproductive technologies are suitable for Kazakh Whiteheaded cattle, aimed at preserving this breed whose population has reduced drastically over the last thirty years. The reproduction characteristics of Kazakh Whiteheaded cows were compared to Aberdeen Angus cows. Transvaginal ultrasound-guided ovum pick up sessions were carried out followed by in vitro embryo production and embryo transfer and pregnancy diagnosis. The total and viable oocytes per OPU procedure were 12.8 ± 1.18 and 8.7 ± 0.85 for the Aberdeen Angus breed, and 8.8 ± 1.04 and 6.2 ± 0.83 for the Kazakh Whiteheaded breed. Similarly, the mean number of cleaved oocytes and morula/blastocyst stage embryos produced by OPU/IVF were 4.8 ± 0.49 and 1.4 ± 0.15 for the Aberdeen Angus breed, and 2.4 ± 0.46 and 0.18 ± 0.05 for the Kazakh Whiteheaded breed (*p* ≤ 0.02). From fifty Kazakh Whiteheaded donor animals, 2585 oocytes were aspirated following six ovum pick up sessions. One thousand eight hundred and seventy-six (72.5%) oocytes were chosen for maturation and were further fertilized. The number of embryos cleaved was 720 (38.3% out of oocytes fertilized) on day four post-fertilization. Of these cleaved embryos, 56 (7.5%) developed into the late morula/blastocyst stage on day seven post-fertilization, averaging 1.12 embryos per donor animal. Pregnancy was detected in 12 recipients; 4 healthy calves have been born to date. The outcomes of our study have demonstrated that reproductive technologies can be applicable in preserving the endangered Kazakh Whiteheaded cattle. The findings in this report will enhance knowledge of the reproductive characteristics of endangered domestic animals and help develop sophisticated reproductive protocols for animals with unique reproductive mechanisms.

## 1. Introduction

In the past 40 years, in vitro embryo production (IVEP) has developed significantly, allowing an increase in the reproductive efficiency of animals with superior genetic merit [1] and the maintenance of the population of endangered species. In vitro fertilization (IVF) has a direct impact on the efficiency of food production and also has great potential for increasing the reproductive efficiency of cattle with economical value [2]. In Kazakhstan, the Kazakh Whiteheaded breed population has decreased dramatically over the last 30 years. The reduction in reproductive efficiency and productivity of dairy and beef animals has mainly been associated with the collapse of the Soviet Union, which resulted in the shutting down of breeding programs, the outflow of specialists and the emergence of transboundary diseases [3,4,5]. The Kazakh Whiteheaded is the main beef breed in Kazakhstan [6] and was bred in the mid-1930s, having high value traits such as disease resistance, 100% unaided calving, thermal adaptation and adaptability to thrive on poor pasture. Kazakh Whiteheaded cattle were obtained by crossing local Kazakh and Kalmyk cows with Hereford bulls in 1950 [7].

These reproductive technologies are of importance in the preservation of endangered wild or domestic species and breeds [8,9]. The application of assisted reproductive technologies (ART) to regain genetically superior and endangered cattle populations in situ and ex situ has been considered for more than thirty years [10,11]. Breeding live animals under in situ management has great potential in conservation biology [12]. However, this conservation method has drawbacks associated with small population size and inbreeding, which can result in genetic drift [12,13] as well as in lower embryo yields and early embryonic death in endangered breeds [14,15]. 

This study aimed to investigate the suitability of IVF technology for the preservation and future propagation of the Kazakh Whiteheaded breed.

## 2. Materials and Methods

### 2.1. Animal Ethics

The experiment was conducted in accordance with national and international laws based on the European Convention for the Protection of Vertebrate Animals used for Experimental and Other Scientific Purposes guidelines [16]. The protocol was approved by the Committee on the Ethics of Animal Experiments of the Saken Seifullin Kazakh Agrotechnical University (permit number: 1607/111).

### 2.2. Cattle

Non-pregnant, cycling (*n* = 50) Kazakh Whiteheaded and Aberdeen Angus (*n* = 50) cows of approximately 48 months of age with high genetic merit were utilized for a species preservation program. The study was conducted from July to October 2020. Animals were maintained on fenced grass (*Agropyron cristatum*) pastures and mineral concentrates were provided ad libitum. Prior to experiment commencement, animals were examined for the presence of any reproductive abnormalities using rectal palpation and ultrasonography. Donor animals were not hormonally prepared for ovum pick up (OPU) sessions according to Pontes, Silva, Basso, Rigo, Ferreira, Santos, Sanches, Porcionato, Vieira, Faifer, Sterza, Schenk, Seneda [17]. Briefly, the rectal contents were removed and the perineal area was thoroughly cleaned with tap water and 70% ethanol. Animals were administered 4 mL of epidural anesthesia 2% lidocaine (Hospira, Inc., Lake Forest, IL, USA) to reduce peristalsis.

### 2.3. Ovum Pickup Procedure (OPU)

Follicular aspiration was carried out according to the protocol described by Gimenes, Ferraz, Fantinato-Neto, Chiaratti, Mesquita, Sá Filho, Meirelles, Trinca, Rennó, Watanabe, Baruselli [18]. Cattle were restrained in a chute and oocyte collection was performed by a single technician using a portable real-time ultrasound scanner (Aloka SSDV 500; Aloka, Tokyo, Japan) with a 7 MHz convex array transducer fitted in a plastic intravaginal probe and a stainless steel guide. Each cow from the given breed was subjected to 6 OPU sessions at 10 day intervals. Visible follicles were punctured using a disposable 16-gauge needle (Supelco Inc., Bellefonte, PA, USA) connected to a 50 mL conical tube (Thermo Fisher Scientific, Waltham, MA, USA) via 2 mm id, 120 cm length silicon circuit tube and aspirated by a vacuum system set at 13–15 mL of water/min. Following aspiration, follicular aspirates were transported to the laboratory for further IVEP manipulations.

### 2.4. In Vitro Embryo Production (IVEP)

Prior to in vitro maturation (IVM), cumulus-oocyte complexes (COC) were morphologically classified utilizing an inverted stereomicroscope according to the protocol based on Leibfried, First [19]. The oocytes with at least one cumulus cell layer were considered grade I and II oocytes and were used for IVF, while cells exhibiting atretic or degradation features were discarded.

Approximately 300 grade I and II oocytes were washed in tissue culture maturation medium (TCM-199- HEPES, Thermo Fisher Scientific, MA, USA) supplemented with 10% fetal calf serum in the presence of antibiotics gentamicin (50 μg/mL), sodium pyruvate (22 mg/mL), and amikacin (83.4 mg/mL). Groups of 30 washed COCs were then cultured for 24 h, 5% CO_2_ in 100 µL drops of IVM medium covered by mineral oil (Sigma–Aldrich, St. Louis, MO, USA). The experiment was run in six replicates and mean values were calculated.

Following IVM, matured COCs were rinsed in PBS and transferred to in vitro fertilization Tyrode-lactate-pyruvate (IVF-TALP) solution (Sigma–Aldrich, St. Louis, MO, USA) supplemented with sodium pyruvate (22 mg/mL), amikacin (83.4 mg/mL), fatty acid-free bovine serum albumin (6 mg/mL), and 70 μL of a solution of PHE (0.5 µM penicillamine, 0.25 µM hypotaurine, and 25 μM epinephrine).

Fresh semen obtained from Kazakh Whiteheaded sires (*n* = 2) was purified in 90–45% Isolate gradient (Irvine Scientific, Santa Ana, CA, USA), diluted in Tyrode medium supplemented with heparin (10 mg/mL), centrifuged at 200× *g* for 30 min, and then examined using AndroVision^®^ software (Minitube, Smythesdale, Australia) for sperm motility and concertation. Ejaculate with final concentration of 2 × 10^6^ live spermatozoa per mL and motility level no less than 90% was used for IVF. Matured oocytes were fertilized in oil-covered 100 µL microdrops in groups of up to 25.

Following IVF, presumptive zygotes had their cumulus cells stripped by washing in TCM-199 HEPES culture medium. Groups of 9–12 presumptive zygotes were then transferred into synthetic oviduct fluid-bovine embryo 2 (SOF-BE2, Sigma, St. Louis, MO, USA) in 100 μL volume micro drops and covered with mineral oil followed by incubation at 39 °C in the presence of 5% CO_2_ in the air. Presumptive zygotes were observed once a day or every other day for the presence of embryo development. Classification of developmental stages of the embryos was carried out in accordance with the International Embryo Transfer Society criteria [20].

### 2.5. Embryo Transfer and Pregnancy Diagnosis

**Figure 1:**
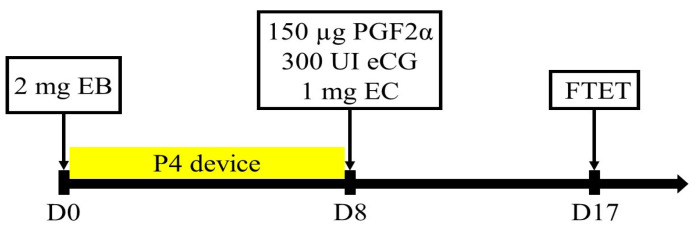
The protocol used for synchronization in recipient animals for fixed-time embryo transfer (FTET). EB: estradiol benzoate, P4: progesterone, eCG: equine chorionic gonadotropin, PGF2α: prostaglandin (d-cloprostenol), and EC: estradiol cypionate.

Nine Kazakh red steppe crossbred cows and four Aberdeen Angus cows were used as surrogates. The experiment was run in triplicates, giving in total 27 and 12 transfers in Kazakh red steppe crossbred cows and Aberdeen Angus cows, respectively. A total of 78 embryos developed into the late morula/blastocyst stage were transferred into the recipient animals. Preparation of recipient animals was performed following a fixed-time embryo transfer (FTET) protocol described by Pontes, Silva, Basso, Rigo, Ferreira, Santos, Sanches, Porcionato, Vieira, Faifer, Sterza, Schenk, Seneda [17], which is shown in Figure 1. The experiment was run in triplicates. For estrus synchronization, on day 0 each cow received an intravaginal progesterone device (CIDR, Pfizer, Auckland, New Zealand) and 2 mg of estradiol benzoate. All cows received 300 IU of equine chorionic gonadotropin (eCG), 150 µg of d-cloprostenol, and 1 mg of estradiol cypionate (Pfizer, New York, NY, USA) on day 8 immediately following progesterone implant removal. Fresh embryos were transplanted on day 17 post estrus synchronization protocol commencement. Each recipient animal received two embryos transferred non-surgically into the uterine horn, ipsilateral to the CL. Recipient animals were thoroughly examined for the presence of corpus luteum (CL) in the ovaries utilizing ultrasonography (Aloka SSD 500 with 7 MHz convex array transducer; Tokyo, Japan). Cows with CL greater or equal to 13 cm in diameter were accepted for ET.

The first session of pregnancy evaluation was performed with ultrasound on days 26–29 following embryo transfer (designated Day 30). Pregnant recipients were subjected to ultrasonography for pregnancy confirmation on day 60 and day 90, respectively.

### 2.6. Data Analysis

Data were analysed with GraphPad Prism, Version 9.0 (San Diego, CA, USA) and Microsoft Excel software. A value of *p* ≤ 0.05 was designated statistically significant. For each variable of interest, descriptive statistics were generated. Data are presented as the mean ± SEM number of oocytes and embryos obtained from donors submitted to OPU/IVP. All variables were assessed using 2-way ANOVA and Sedak multiple comparison tests to define significant differences between groups.

## 3. Results

In vitro fertilization data obtained from Kazakh Whiteheaded preservation experiments are demonstrated in comparison to Aberdeen Angus breed in Table 1. In total, 2585 and 3854 oocytes were aspirated from Kazakh Whiteheaded and Aberdeen Angus donor animals. Each cow was exposed to six OPU sessions at 10-day intervals.

The mean number of recovered oocytes (12.8 ± 1.18 vs. 8.8 ± 1.04), viable oocytes (8.7 ± 0.85 vs. 6.2 ± 0.83), cleaved oocyte yield (4.8 ± 0.49 vs. 2.4 ± 0.46), and embryos that reached Morula/blastocyst stage (1.4 ± 0.15 vs. 0.18 ± 0.05) per procedure was significantly greater (*p* ≤ 0.02) in the Aberdeen Angus than in the Kazakh Whiteheaded cows.

**Figure 2:**
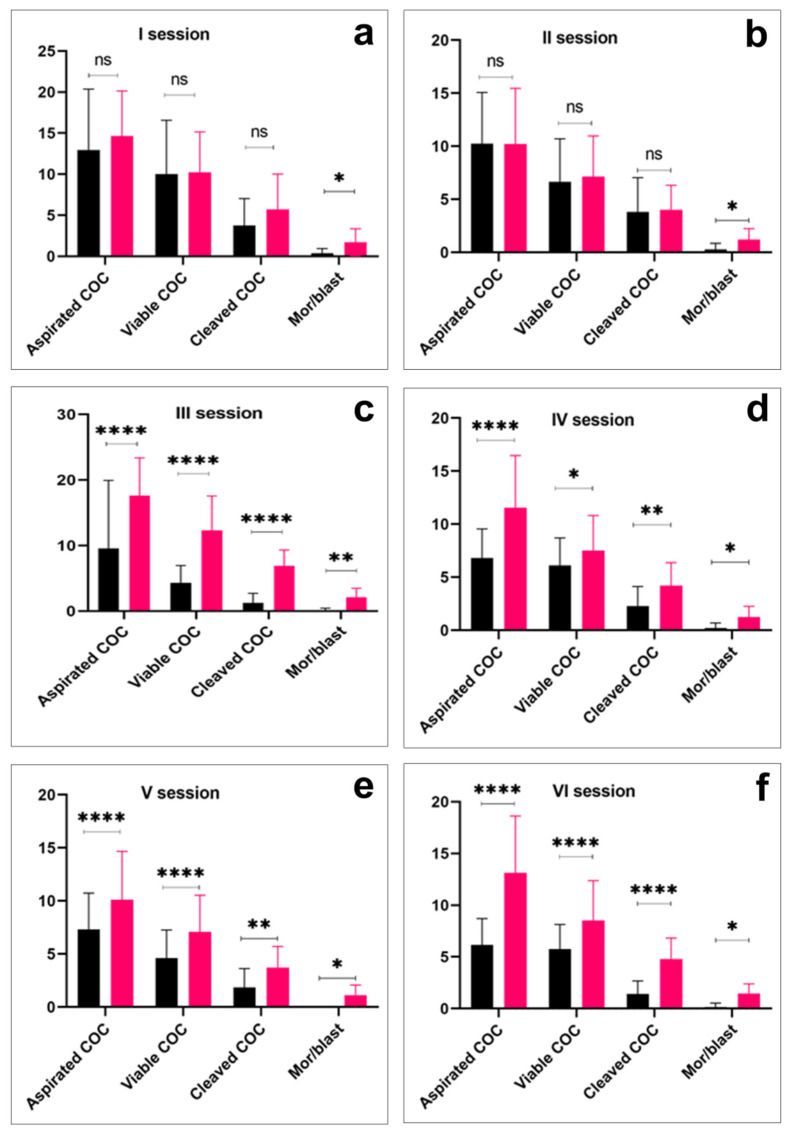
Per-session comparison of the number of cumulus oocyte complexes (COC) aspiration, viable COC, cleaved COC, and embryo production efficiency in Kazakh Whiteheaded (black bars) and Aberdeen Angus breeds (red bars). Asterisk over bars demonstrated degree of significance, where * *p* < 0.05; ** *p* < 0.01; *** *p* < 0.001; **** *p* < 0.0001; ns = not significant.

Overall, in six OPU sessions, the total number of recovered COCs, the number of viable oocytes, the number of cleaved oocytes, and the morula/blastocyst ratio were higher in the Aberdeen Angus group (*p* < 0.01) than in the Kazakh Whiteheaded group (Table 1). However, the overall proportion of viable oocytes was higher in the Kazakh Whiteheaded (1876/2585 (72.5%)) than in the Aberdeen Angus donor animals (2642/3864 (68.3%)). Additionally, in per-session comparison, almost all variables were significantly higher in the Aberdeen Angus group (*p* < 0.0001) than in the Kazakh Whiteheaded group (Figure 2). 

In Kazakh Whiteheaded cows, the number of oocytes acquired per cow OPU session varied from 3 to 22. Viable oocytes were evaluated for maturation and were subsequently fertilized. The number of embryos cleaved was 720 (38.3% out of oocytes fertilized) on day 4 post-fertilization. Of these cleaved embryos, 56 (7.5%) developed into the late morula/blastocyst stage on day 7 post-fertilization, averaging 1.12 (2%) embryos per donor animal. Twelve recipients were diagnosed pregnant on day 30 post-fertilization by ultrasonography whereas, on day 90, pregnancy was confirmed in five cows. Four calves were successfully delivered by the end of the gestation period, whereas one cow aborted in the late second trimester.

In Aberdeen Angus cows, nine recipients were diagnosed pregnant on day 30 post-fertilization by ultrasonography. These animals carried their full-term pregnancies and delivered live calves by the end of gestation.

## 4. Discussion

The use of in vitro fertilization technology has become popular and has been in high demand worldwide following the successful birth of the first IVF calf in 1981 [21]. To our knowledge, this is the first attempt at using IVF technology in Kazakh Whietheaded cattle. In recent years, the embryo transfer industry has achieved significant results in improving livestock production [22].

The application of the ex situ program for the conservation of oocytes, spermatozoa, and embryos has a significant advantage, allowing their development to be resumed at the desired time [23,24]. Contreras, Galina, and Chenoweth [25] reported that cryoconservation has a low-efficiency due to the high risk of damaging reproductive materials. For this reason, our initial research mainly focused on the production of embryos and birth rates. However, the importance of cryoconservation on Kazakh Whiteheaded embryos remains relevant and needs to be evaluated in future experiments.

On the contrary, applying current improvements in conservation biology, the semen of desired sires can be stored using the vitrification method [26]. Even though semen conservation has cost- and quality-related benefits, there are limitations related to herd regeneration, as it demands time-consuming evaluation of another breed over several generations by backcrossing [10]. In line with these considerations, the preservation of threatened breeds using embryos is promising and one generation is adequate to regain breeding [27]. Due to a scarcity of literature on Kazakh Whiteheaded cows, their response to in vivo and in vitro ET needs to be further investigated. Initially, for embryo production, the MOET protocol was used based on Pontes, Nonato-Junior, Sanches, Ereno-Junior, Uvo, Barreiros, Oliveira, Hasler, and Seneda [28]. However, this method has been inefficient for inducing superovulation due to a meagre response to treatment and therefore was not used for embryo collection. The superovulation protocol suitable for the Kazakh Whiteheaded cow is under evaluation.

In Kazakh Whiteheaded cows, the average number of transferable embryos per animal was lower than those published by Ratto, Peralta, Mogollon, Strobel, Correa [29], Pontes, Silva, Basso, Rigo, Ferreira, Santos, Sanches, Porcionato, Vieira, Faifer, Sterza, Schenk, and Seneda [17], and Bousquet, Twagiramungu, Morin, Brisson, Carboneau, and Durocher [30], accounting for 1.12, 1.8, 2.1, and 4.7, respectively. This could be explained by the large oocytes yield due to the application of exogenous hormones in their study. However, this was not evaluated in our study. In the present study, variables of the first two OPU sessions had not differed between breeds. However, we observed a significant decrease in the number of oocytes obtained in the following OPU session in Kazakh Whiteheaded cows. A similar pattern was observed in Aberdeen Angus groups, with a slight decrease. This observation is in agreement with the finding reported by Monteiro, Batista, Vieira, Bayeux, Accorsi, Campanholi, Dias, Souza, and Baruselli [31]. Viana [32] reported that a decrease in the number of oocytes aspirated could be because of repeatable ovarian punctures with a needle during consecutive OPU. They assume that multiple punctures performed in animals with many follicular developments may result in ovarian damage and weaken its performance.

The average number of oocytes recovered per OPU session (*n* = 8.8) from Kazakh Whiteheaded cows was lower than that recovered from the Aberdeen Angus breed (12.8) and those reported by Pontes, Silva, Basso, Rigo, Ferreira, Santos, Sanches, Porcionato, Vieira, Faifer, Sterza, Schenk, and Seneda [17] and Viana [33]. These authors reported collecting on average 17.1 and 18.8 oocytes per OPU session, respectively. Additionally, the highest number of viable oocytes per OPU session in Kazakh Whiteheaded cows constituted (*n* = 10) cells per animal, which is peculiar in comparison to those published by Pontes, Silva, Basso, Rigo, Ferreira, Santos, Sanches, Porcionato, Vieira, Faifer, Sterza, Schenk, and Seneda [17]. In their large-scale in vitro embryo production program, Gir cows yielded (*n* = 12.1) viable oocytes per OPU session.

On the contrary, the average proportion of viable oocytes was higher in Kazakh Whiteheaded cows (73.5%) when compared to the Aberdeen Angus breed (67.8%) and others to Gir (70.9%), Nellore (68.6%), and Holstein (53.8%) breeds, respectively [17,18]. Moreover, other large-scale commercial programs using Nellore cattle reported a similar result to our study, demonstrating a viable oocytes rate of 75% [34]. Furthermore, the cleavage and blastocyst rates of the Kazakh Whiteheaded cattle (Table 1) are significantly lower than those of the Nellore and Holstein cattle ^18^.

In general, we demonstrated some similarities with the other works mentioned above, except for inferior cleavage and blastocyst rates, which resulted in a low birth rate in Kazakh Whiteheaded cows.

In this study, we have demonstrated that Kazakh Whiteheaded cows had lower COC production rates, IVP efficiency, and morula/blastocyst development rates in comparison to Aberdeen Angus cows. These could be explained by the potential genetics of the breed [29] and potential influence of hybrid vigor on the embryos from Angus cows compared to the purebred embryos from Kazakh Whitehead cows.

As we mentioned earlier in this paper, no references were available regarding IVF application in Kazakh Whiteheaded cattle. Considering that the pregnancy rate was low concerning the number of aspirated oocytes, we infer that this phenomenon may be a peculiarity of Kazakh Whiteheaded cattle; however, due to the importance of the breed, further research must be carried out to improve IVEP rates. Although the efficiency of reproductive biotechnologies may vary significantly and is generally considered low [10], the method itself plays a crucial role for species recovery programs, and for long-term genetic and demographic sustainability [11,35].

## Figures and Tables

**Table 1:** Data obtained from IVF experiment in Kazakh Whiteheaded and Aberdeen Angus breeds.

Variables	Kazakh Whiteheaded Breed, OPU Sessions, (*n* = 50 Individuals)						Aberdeen Angus Breed, OPU Sessions, (*n* = 50 Individuals)						Mean ± SEM Number of Oocytes and Embryos Obtained
	1	2	3	4	5	6	1	2	3	4	5	6	
Number of oocytes aspirated/mean per animal	646/ 12.9 ± 7.4	512/ 10.2 ± 4.8	412/ 9.5 ± 10.3	341/ 6.8 ± 2.7	366/ 7.3 ± 3.4	308/ 6.1 ± 2.5	732/ 14.6 ± 5.5	510/ 10.2 ± 5.2	882/ 17.6 ± 5.7 *	577/ 11.5 ± 4.9 *	506/ 10.1 ± 4.5 *	657/ 13.1 ± 5.4 *	21.6 ± 1.1 6449
Number of viable oocytes/mean per animal	503/ 10 ± 6.5	333/ 6.6 ± 4.0	215/ 4.3 ± 2.6	306/ 6.1 ± 2.5	231/ 4.6 ± 2.6	288/ 5.7 ± 2.3	512/ 10.24 ± 4.9	357/ 7.14 ± 3.8	617/ 12.3 ± 5.2 *	375/ 7.5 ± 3.3 *	354/ 7.08 ± 3.4 *	427/ 8.5 ± 3.8 *	14.9 ± 2.7 4518
Number of cleaved oocytes/mean per animal	188/ 3.7 ± 3.2	191/ 3.8 ± 3.2	63/ 1.2 ± 1.4	114/ 2.2 ± 1.8	93/ 1.8 ± 1.7	71/ 1.4 ± 1.2	286/ 5.72 ± 4.3	200/ 4.00 ± 2.3	346/ 6.9 ± 2.4 *	210/ 4.2 ± 2.1 *	186/ 3.7 ± 1.9 *	239/ 4.7 ± 2.0 *	7.2 ± 0.8 2187
Morula/blastocyst stage embryos on day 7	18/ 0.3 ± 0.5	14/ 0.2 ± 0.5	6/ 0.1 ± 0.3	11/ 0.2 ± 0.4	0/ 0.00 ± 0.00	7/ 0.1 ± 0.4	86/ 1.72 ± 1.6 *	60/ 1.2 ± 1.0 *	104/ 2.0 ± 1.4 *	63/ 1.2 ± 1.0 *	56/ 1.1 ± 0.9 *	72/ 1.4 ± 0.9 *	1.58 ± 0.08 497

* 2-way ANOVA and Sedak multiple comparison tests.

## Data Availability

Not applicable.

## References

[1] Hasler J.F. (2014). Forty years of embryo transfer in cattle: A review focusing on the journal Theriogenology, the growth of the industry in North America, and personal reminisces. Theriogenology.

[2] Hansen P.J., Lamb G.C., DiLorenzo N. (2014). Current and Future Assisted Reproductive Technologies for Mammalian Farm Animals. Current and Future Reproductive Technologies and World Food Production.

[3] AQBAS P. (2012). Republican Association of the Kazakh Whiteheaded Breed. https://eldala.kz/dannye/kompanii/620-respublikanskaya-palata-kazahskoj-belogolovoj-porody.

[4] Issimov A., Kushaliyev K., Abekeshev N., Molla W., Rametov N., Bayantassova S., Zhanabayev A., Paritova A., Shalmenov M., Ussenbayev A. (2022). Risk factors associated with lumpy skin disease in cattle in West Kazakhstan. Prev. Veter. Med..

[5] Bayantassova S., Kushaliyev K., Zhubantayev I., Zhanabayev A., Kenzhegaliyev Z., Ussenbayev A., Paritova A., Baikadamova G., Bakishev T., Zukhra A. (2023). Knowledge, attitude and practice (KAP) of smallholder farmers on foot-and-mouth disease in Cattle in West Kazakhstan. Vet. Med. Sci..

[6] Issimov A., Baibatyrov T., Tayeva A., Kenenbay S., Abzhanova S., Shambulova G., Kuzembayeva G., Kozhakhiyeva M., Brel-Kisseleva I., Safronova O. (2022). Prevalence of *Clostridium perfringens* and Detection of Its Toxins in Meat Products in Selected Areas of West Kazakhstan. Agriculture.

[7] Kineev M.A., Erdenov B.K. (2005). Cattle Breeds of Kazakhstan. Research and Production Center for Animal Husbandry and Veterinary Medicine.

[8] Herrick J.R. (2019). Assisted reproductive technologies for endangered species conservation: Developing sophisticated protocols with limited access to animals with unique reproductive mechanisms. Biol. Reprod..

[9] Hall S.J.G., Brenig B., Ashdown R.A., Curry M.R. (2021). Conservation of rare wild-living cattle *Bos taurus* (L.): Coat colour gene illuminates breed history, and associated reproductive anomalies have not reduced herd fertility. J. Zool..

[10] Varga Z., Bárádi Z., Macháty Z., Solti L., Cseh S., Seregi J., Csáki T., Vajta G. (1993). In-vitro fertilization in hungarian gray cattle. Reprod. Domest. Anim..

[11] Mastromonaco G.F., Gonzalez-Grajales A.L. (2020). Reproduction in female wild cattle: Influence of seasonality on ARTs. Theriogenology.

[12] Dolman P.M., Collar N.J., Scotland K.M., Burnside R.J. (2015). Ark or park: The need to predict relative effectiveness of ex situ and in situ conservation before attempting captive breeding. J. Appl. Ecol..

[13] Tada O., Muchenje V., Dzama K. (2013). Short communication: Effective population size and inbreeding rate of indigenous Nguni cattle under in situ conservation in the low-input communal production system. S. Afr. J. Anim. Sci..

[14] Mastromonaco G.F., Coppola G., Crawshaw G., DiBerardino D., King W.A. (2004). Identification of the homologue of the bovine Rob(1;29) in a captive gaur (*Bos gaurus*). Chromosom. Res..

[15] Hintz R.L., Foose T.J. (1982). Inbreeding, mortality, and sex ratio in gaur (*Bos gaurus*) under captivity. J. Hered..

[16] Ausems E.J. (1986). The european convention for the protection of vertebrate animals used for experimental and other scientific purposes. Z. Fur Vers..

[17] Pontes J., Silva K., Basso A., Rigo A., Ferreira C., Santos G., Sanches B., Porcionato J., Vieira P., Faifer F. (2010). Large-scale in vitro embryo production and pregnancy rates from *Bos taurus*, *Bos indicus*, and indicus-taurus dairy cows using sexed sperm. Theriogenology.

[18] Gimenes L.U., Ferraz M.L., Fantinato-Neto P., Chiaratti M.R., Mesquita L.G., Filho M.F.S., Meirelles F.V., Trinca L.A., Rennó F.P., Watanabe Y.F. (2015). The interval between the emergence of pharmacologically synchronized ovarian follicular waves and ovum pickup does not significantly affect in vitro embryo production in *Bos indicus*, *Bos taurus*, and *Bubalus bubalis*. Theriogenology.

[19] Leibfried L., First N.L. (1979). Characterization of Bovine Follicular Oocytes and Their Ability to Mature In Vitro. J. Anim. Sci..

[20] Wright J.M. (1998). Photographic illustrations of embryo developmental stage and quality codes. Man. Int. Embryo Transf. Society.

[21] Brackett B.G., Bousquet D., Boice M.L., Donawick W.J., Evans J.F., Dressel M.A. (1982). Normal Development Following In Vitro Fertilization in the Cow. Biol. Reprod..

[22] Currin L., Baldassarre H., Bordignon V. (2021). In Vitro Production of Embryos from Prepubertal Holstein Cattle and Mediterranean Water Buffalo: Problems, Progress and Potential. Animals.

[23] Rusciano G., De Canditiis C., Zito G., Rubessa M., Roca M.S., Carotenuto R., Sasso A., Gasparrini B. (2017). Raman-microscopy investigation of vitrification-induced structural damages in mature bovine oocytes. PLoS ONE.

[24] Gororo E., Chatiza F.P., Chidzwondo F., Makuza S.M. (2021). Is neutral genetic diversity related to quantitative variation in semen traits in bulls?. Reprod. Domest. Anim..

[25] Contreras D.A., Galina C.S., Chenoweth P. (2021). Prospects for increasing the utilization of cattle embryo transfer by small-scale milk and meat producers in tropical regions. Reprod. Domest. Anim..

[26] Grötter L.G., Cattaneo L., Marini P.E., Kjelland M.E., Ferré L.B. (2019). Recent advances in bovine sperm cryopreservation techniques with a focus on sperm post-thaw quality optimization. Reprod. Domest. Anim..

[27] Iso-Touru T., Tapio M., Vilkki J., Kiseleva T., Ammosov I., Ivanova Z., Popov R., Ozerov M., Kantanen J. (2016). Genetic diversity and genomic signatures of selection among cattle breeds from Siberia, eastern and northern Europe. Anim. Genet..

[28] Pontes J., Nonato-Junior I., Sanches B., Ereno-Junior J., Uvo S., Barreiros T., Oliveira J., Hasler J., Seneda M. (2009). Comparison of embryo yield and pregnancy rate between in vivo and in vitro methods in the same Nelore (*Bos indicus*) donor cows. Theriogenology.

[29] Ratto M., Peralta O., Mogollon G., Strobel P., Correa J. (2011). Transvaginal ultrasound-guided cumulus oocyte complexes aspiration and in vitro embryo production in suckled beef and lactating dairy cattle on pasture-based management conditions. Anim. Reprod. Sci..

[30] Bousquet D., Twagiramungu H., Morin N., Brisson C., Carboneau G., Durocher J. (1999). In vitro embryo production in the cow: An effective alternative to the conventional embryo production approach. Theriogenology.

[31] Monteiro F., Batista E., Vieira L., Bayeux B., Accorsi M., Campanholi S., Dias E., Souza A., Baruselli P. (2016). Beef donor cows with high number of retrieved COC produce more in vitro embryos compared with cows with low number of COC after repeated ovum pick-up sessions. Theriogenology.

[32] Viana J.H.M., Nascimento A.A., Pinheiro N.L., Ferreira A.M., Camargo L.S., Sá W.F., Marques Júnior A.P. (2003). Characterization of tissue damages after ovum pick-up in bovine. Pesqui. Veterinária Bras..

[33] Viana J.H.M., Palhão M.P., Arashiro E.K.N., Ferreira A.M., Fonseca J.F., Fernandes C.A.C. (2004). Pre-synchronization of cows for cumulusoocyte complexes recover: Partial results. Acta Sci. Vet..

[34] Pontes J., Sterza F.M., Basso A., Ferreira C., Sanches B., Rubin K., Seneda M. (2011). Ovum pick up, in vitro embryo production, and pregnancy rates from a large-scale commercial program using Nelore cattle (*Bos indicus*) donors. Theriogenology.

[35] Ralls K., Ballou J. (1986). Captive breeding programs for populations with a small number of founders. Trends Ecol. Evol..

